# Supplementary material for: Developing contents for a digital adherence tool: A formative mixed-methods study among children and adolescents living with HIV in Tanzania
Source: PLOS Digit Health. 2023 Oct 18;2(10):e0000232. doi: 10.1371/journal.pdig.0000232 (PMC10584100; doi:10.1371/journal.pdig.0000232)
Supplement: S1 Appendix — (DOCX) [file pdig.0000232.s001.docx]

**S1 Appendix: Characteristics of children in the DAT intervention (N=20)**

*Self-report Adherence = How many pills were not swallowed in the past month*

*Pharmacy refill adherence = (Pills provided in the previous visit - left overs)/ (number of days between pharmacy visits) x 100%*

*DAT adherence = (number of intakes)/ (number of days the device was in use) x 100%*

| Sex | Age range | Years in HIV care | Self-reported  Adherence (%) | Pharmacy refill  Adherence (%) | DAT  Adherence (%) |
| --- | --- | --- | --- | --- | --- |
| Male | 6-10 | 7 | 100 | 100 | 96 |
| Male | 11-15 | 9 | 100 | 79 | 100 |
| Male | 11-15 | 10 | 100 | 100 | 85 |
| Male | 6-10 | 7 | 100 | 82 | 97 |
| Female | 6-10 | 4 | 100 | 100 | 100 |
| Male | 11-15 | 0 | 100 | 97 | 100 |
| Male | 0-5 | 1 | 100 | 100 | 100 |
| Female | 11-15 | 9 | 100 | 100 | 100 |
| Male | 11-15 | 9 | 100 | 100 | 100 |
| Female | 6-10 | 7 | 100 | 42 | 100 |
| Female | 6-10 | 2 | 100 | 42 | 96 |
| Female | 11-15 | 3 | 100 | 100 | 100 |
| Male | 11-15 | 9 | 100 | 10 | 96 |
| Female | 6-10 | 5 | 100 | 80 | 80 |
| Male | 11-15 | 9 | 100 | 100 | 97 |
| Male | 6-10 | Unknown | 100 | 100 | 08 |
| Female | 6-10 | 10 | 100 | 91 | 32 |
| Female | 6-10 | 3 | 100 | 86 | 100 |
| Female | 0-5 | 0 | 100 | 100 | 100 |
| Male | 11-15 | 9 | 67 | 96 | 73 |
| Median (IQR) | 10(7.2-11.8) | 7(2.2-9) | 100(100-100) | 98.4(80.5-100) | 98.5(87.7-100) |
