# Supplementary material for: Developing contents for a digital adherence tool: A formative mixed-methods study among children and adolescents living with HIV in Tanzania
Source: PLOS Digit Health. 2023 Oct 18;2(10):e0000232. doi: 10.1371/journal.pdig.0000232 (PMC10584100; doi:10.1371/journal.pdig.0000232)
Supplement: S2 Appendix — (DOCX) [file pdig.0000232.s002.docx]

**S2 Appendix: Characteristics of adolescents in the DAT intervention (N=20)**

*Self-report Adherence = How many pills were not swallowed in the past month*

*Pharmacy refill adherence = (Pills provided in the previous visit - left overs)/ (number of days between pharmacy visits) x 100%*

*DAT adherence = (number of intakes)/ (number of days the device was in use) x 100%*

| **Sex** | **Age range** | **Years in HIV care** | **Self-reported Adherence (%)** | **Pharmacy refill Adherence (%)** | **DAT Adherence (%)** |
| --- | --- | --- | --- | --- | --- |
| Male | 15-19 | 6 | 93 | 98 | 0 |
| Female | 15-19 | 17 | 97 | 82 | 20 |
| Female | 15-19 | 12 | 100 | 92 | 56 |
| Female | 15-19 | 17 | 100 | 53 | 47 |
| Male | 15-19 | 16 | 90 | 87 | 24 |
| Male | 15-19 | 11 | 90 | 90 | 20 |
| Female | 15-19 | 10 | 100 | 100 | 92 |
| Male | 15-19 | 15 | 93 | 79 | 4 |
| Male | 15-19 | 06 | 100 | 97 | 100 |
| Female | 15-19 | 16 | 100 | 99 | 79 |
| Female | 15-19 | 12 | 100 | 100 | 50 |
| Male | 15-19 | 10 | 100 | 97 | 100 |
| Male | 15-19 | 08 | 100 | 100 | 84 |
| Male | 15-19 | 16 | 87 | 100 | 96 |
| Male | 15-19 | 12 | 100 | 100 | 67 |
| Female | 15-19 | 12 | 100 | 100 | 25 |
| Male | 15-19 | 13 | 77 | 100 | 76 |
| Male | 15-19 | 13 | 100 | 97 | 88 |
| Male | 15-19 | 10 | 100 | 90 | 89 |
| Female | 15-19 | 12 | 100 | 94 | 100 |
| Median (IQR) | 18(18-18) | 12(10-15.8) | 100(93.3-100) | 96.9(90-100) | 72(24.2-91.2) |
