# Supplementary material for: Developing contents for a digital adherence tool: A formative mixed-methods study among children and adolescents living with HIV in Tanzania
Source: PLOS Digit Health. 2023 Oct 18;2(10):e0000232. doi: 10.1371/journal.pdig.0000232 (PMC10584100; doi:10.1371/journal.pdig.0000232)
Supplement: S3 Appendix — (DOCX) [file pdig.0000232.s003.docx]

**S3 Appendix: Adolescents SMS Preference (N=20)**

|  | SMS contents | YES (%) | No (%) | Do not remember the SMS (%) |
| --- | --- | --- | --- | --- |
| WEEK1 | "Hello, your time to take the medication is near, you are reminded to take your medication on time as directed by the health care workers” | **13(65%)** | 6(30%) | 1(5%) |
| WEEK2 | **"**Remember to observe your health."  **"**Remember to protect your health today."  "Don’t stop caring for your health today"  “Your time is at hand"?  **"**You are reminded to protect your health"  "Remember to drink on time"?  "Your time to use is at hand" | 16(80%)  16(80%)  18(90%)  15(75%)  17(85%)  15(75%)  12(60%) | 3(15%)  3(15%)  1(5%)  4(20%)  2(10%)  4(20%)  6(30%) | 1(5%)  1(5%)  1(5%)  1(5%)  1(5%)  1(5%)  2(10%) |
| WEEK3 | "You are reminded to drink”?  "Do not forget to use”?  "The time is at hand"?  "Your health is important”  "Do not forget to protect yourself"  **"**Care for your health”  "Drinking is caring”! | 14(70%)  16(80%)  19(95%)  18(90%)  18(90%)  19(95%)  9(45%) | 4(20%)  2(10%)  0(0%)  0(0%)  1(5%)  1(5%)  4(20%) | 2(10%)  2(10%)  1(5%)  2(10%)  1(5%)  0(0%)  7(35%) |
| WEEK4 | "Remember"?  "Use”!  "Take care"?  "Drink"?  "Health"  "Care for your health"  "Value health"? | 13(65%)  13(65%)  15(75%)  15(75%)  15(75%)  18(90%)  15(75%) | 1(5%)  2(10%)  1(5%)  4(20%)  1(5%)  1(5%)  1(5%) | 6(30%)  5(25%)  4(20%)  1(5%)  4(20%)  1(5%)  4(20%) |
