# Supplementary material for: Developing contents for a digital adherence tool: A formative mixed-methods study among children and adolescents living with HIV in Tanzania
Source: PLOS Digit Health. 2023 Oct 18;2(10):e0000232. doi: 10.1371/journal.pdig.0000232 (PMC10584100; doi:10.1371/journal.pdig.0000232)
Supplement: S4 Appendix — (DOCX) [file pdig.0000232.s004.docx]

**S4 Appendix: Children SMS Preference (N=20)**

(Questions were answered by caregivers/parent)

| WEEKS | SMS contents | YES (%) | No (%) | | | Did not remember the SMS (%) |
| --- | --- | --- | --- | --- | --- | --- |
| WEEK1 | "Hello, you are reminded that the time for your child to take medication is approaching. Kindly give the child the medication on time as instructed by the health care workers. Thank you. | 12(60%) | 8(40%) | | 0(0%) | |
| WEEK2 | "Remember to observe child’s health”  "Kindly, remember to protect the child’s health today”  "Don’t stop to care for the child’s health”  **"** The time for the child is approaching”  "You are reminded to protect child’s health”  "Remember to give the child on time”  "The time for the child to use is at hand” | 17(85%)  16(80%)  16(80%)  16(80%)  16(80%)  16(80%)  18(90%) | | 1(5%)  1(5%)  1(5%)  2(10%)  2(10%)  2(10%)  1(5%) | 2(10%)  3(15%)  3(15%)  2(10%)  2(10%)  2(10%)  1(5%) | |
| WEEK3 | "You are reminded to feed the child”  "Don’t forget to give the child”  “Child’s time is approaching”  "Health of the child is important”  "Don't stop to protect the child”  "Care for child’s health”  **"**Feeding the child is the act of caring” | 13(65%)  15(75%)  16(80%)  18(90%)  17(85%)  17(85%)  15(75%) | | 3(15%)  3(15%)  2(10%)  1(5%)  1(5%)  1(5%)  2(10%) | 4(20%)  2(10%)  2(10%)  1(5%)  2(10%)  2(10%)  3(15%) | |
| WEEK4 | “Remember the child”  "Give the child."?  "Protect the child”  "Feed the child"?  "Care for child’s health"?  “Protect child’s health”  “ Child’s health” | 15(75%)  13(65%)  12(60%)  16(80%)  16(80%)  17(85%)  16(80%) | | 1(5%)  3(15%)  4(20%)  0(0%)  1(5%)  1(5%)  0(0%) | 4(20%)  4(20%)  4(20%)  4(20%)  3(15%)  2(10%)  4(20%) | |
