# Supplementary material for: Developing contents for a digital adherence tool: A formative mixed-methods study among children and adolescents living with HIV in Tanzania
Source: PLOS Digit Health. 2023 Oct 18;2(10):e0000232. doi: 10.1371/journal.pdig.0000232 (PMC10584100; doi:10.1371/journal.pdig.0000232)

**S5 Appendix: Example of an adherence feedback graph.**

*DAT adherence = (number of intakes)/ (number of days the device was in use) x 100%*


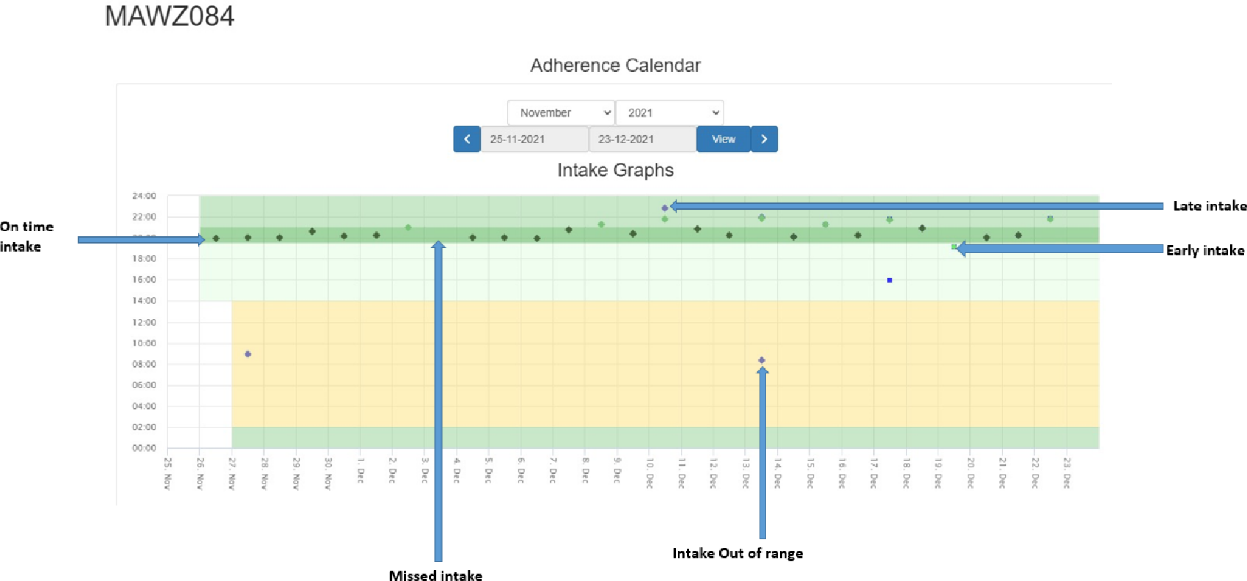

Supplement: S5 Appendix — (DOCX) [file pdig.0000232.s005.docx]
