# Supplementary material for: Developing contents for a digital adherence tool: A formative mixed-methods study among children and adolescents living with HIV in Tanzania
Source: PLOS Digit Health. 2023 Oct 18;2(10):e0000232. doi: 10.1371/journal.pdig.0000232 (PMC10584100; doi:10.1371/journal.pdig.0000232)
Supplement: Attachment — Submitted filename: Response_to_reviewers.pdf [file pdig.0000232.s006.pdf]

Dear reviewer,

We are grateful for the opportunity to revise the manuscript. The comments from the reviewers are genuine and will really assist us in improving the manuscript for publication.

Please, find a response to each comment point-by-point below:

Journal Requirements:

1. Please amend your detailed Financial Disclosure statement. This is published with the article. It must therefore be completed in full sentences and contain the exact wording you wish to be published.

We thank the editor for this comment on the requirement of the financial disclosure statement. We have amended the financial disclosure statement to include the required details.

a. State the initials, alongside each funding source, of each author to receive each grant.

The whole project was funded through one source, which is the European and Developing Countries Clinical Trials Partnership (EDCTP) under the senior fellowship plus TMA2818. We mentioned this in the Financial Disclosure statement.

b. State what role the funders took in the study. If the funders had no role in your study, please state: "The funders had no role in study design, data collection and analysis, decision to publish, or preparation of the manuscript."

We have added the following statement to the financial disclosure statement 'The funders had no role in study design, data collection and analysis, decision to publish, or preparation of the manuscript.'

c. If any authors received a salary from any of your funders, please state which authors and which funders.

None of the authors received direct salary from the funder. The whole project, including human resources, was supported by EDCTP under the senior fellowship plus TMA2818 awarded to MSB.

If you did not receive any funding for this study, please simply state: "The authors received no specific funding for this work."

This is not applicable to us as we received funding for the study.

2. We do not publish any copyright or trademark symbols that usually accompany proprietary names, eg ©, ®, ™ (e.g. next to drug or reagent names). Please remove all instances of trademark/copyright symbols throughout the text, including ® on pages 2, 8, 9, 10 and 15.

We thank the editor for this comment. We have done accordingly all over the manuscript.

3. We ask that a manuscript source file is provided at Revision. Please upload your manuscript file as a .doc, .docx, .rtf or .tex.

This has been noted, we will upload the manuscript file in the required format.

4. In the online submission form, you indicated that "The quantitative and qualitative data used in this study will be made available upon request from the corresponding author". All PLOS journals now require all data underlying the findings described in their manuscript to be freely available to other researchers, either 1. In a public repository, 2. Within the manuscript itself, or 3. Uploaded as supplementary information.
This policy applies to all data except where public deposition would breach compliance with the protocol approved by your research ethics board. If your data cannot be made publicly available for ethical or legal reasons (e.g., public availability would compromise patient privacy), please explain your reasons by return email and your exemption request will be escalated to the editor for approval. Your exemption request will be handled independently and will not hold up the peer review process, but will need to be resolved should your manuscript be accepted for publication. One of the Editorial team will then be in touch if there are any issues.

We understand the importance of open-access. Though currently, our data cannot be made publicly as the regulations in Tanzania do not allow for data sharing without arranging a data transfer agreement with the receiver of the data. During submission we have stated that data will be available upon request. We will, then, have to follow the Tanzanian law for data-sharing and the authorities will have to approve the data-sharing request by approving a data transfer agreement. Currently, Tanzania is developing their own data repository where meta-data of our study will be shared soon.

Reviewers' comments:

Reviewer's Responses to Questions

1. Does this manuscript meet PLOS Digital Health’s publication criteria? Is the manuscript technically sound, and do the data support the conclusions? The manuscript must describe methodologically and ethically rigorous research with conclusions that are appropriately drawn based on the data presented

Reviewer #1: Yes

Reviewer #2: Partly

We thank the reviewer for checking the publication criteria and whether it is technically sound and data supports our conclusions. In addition, to see whether it describes methodologically and ethically rigorous research. We will

respond to specific queries below.

2. Has the statistical analysis been performed appropriately and rigorously?

Reviewer #1: Yes

Reviewer #2: I don't know

We thank the reviewer 1 for positively judging the statistical analysis.

3. Have the authors made all data underlying the findings in their manuscript fully available (please refer to the Data Availability Statement at the start of the manuscript PDF file. PLOS Data policy requires authors to make all data underlying the findings described in their manuscript fully available without restriction, with rare exception. The data should be provided as part of the manuscript or its supporting information, or deposited to a public repository. For example, in addition to summary statistics, the data points behind means, medians and variance measures should be available. If there are restrictions on publicly sharing data—e.g. participant privacy or use of data from a third party—those must be specified.

Reviewer #1: Yes

Reviewer #2: Yes

We thank the reviewer for positively judging the availability of data. During submission, we have stated that data will be available upon request. We will, then, have to follow the Tanzanian law for data-sharing, and the authorities will have to approve the data-sharing request by approving a data transfer agreement. Currently, Tanzania is developing their data repository where the meta-data of our study will be shared soon.

4. Is the manuscript presented in an intelligible fashion and written in standard English? PLOS Digital Health does not copyedit accepted manuscripts, so the language in submitted articles must be clear, correct, and unambiguous. Any typographical or grammatical errors should be corrected at revision, so please note any specific errors here.

Reviewer #1: Yes

Reviewer #2: No

We thank the reviewers for checking the English standard writing. We will respond to specific queries below.

5. Review Comments to the Author.

Reviewer #1: General;
Title: excessive length-difficult to determine primary objective/endpoint and intervention.

We thank the reviewer for this valuable comment. We have changed the title to 'Developing contents for a digital adherence tool: a formative mixed-methods study among children and adolescents living with HIV in Tanzania'

Formatting: line numbering would greatly facilitate review by locating content more easily. Therefore, specific comment locations are restricted to section/page references.

We have added line numbers to the document accordingly.

Paragraph spacing inconsistent throughout.

Thanks for this observation. We have corrected this throughout the manuscript.

Inclusion of patient quotes not helpful, since uncertain how it was chosen (consistent and representative of others or contrasting).

We thank the reviewer for this comment. Though, we do not fully agree with the reviewer as this is a mixed-methods study design whereby the display of quotes gives more insight into the context of the quantitative results. Qualitative data analyses are based on quotes from narratives and the most illustrative ones are chosen to be displayed. We realize that there always is some level of subjectivity in the choice of quotes, but that is inherent to qualitative results displayed. Therefore, to reduce length we have decided to remove the quotes in the manuscript body and include them in table 2 instead.

Abstract: Needs significant revision/reorganization, since it is difficult to follow. See author instructions to assist with organization. Background/objective/population/intervention/endpoint analysis/results/significance would be easier to follow.

We thank the reviewer for these very important observations. We have restructured and rewritten the abstract according to the comments of the reviewer. However, it was not very clear to us if all the comments under this section refer to the abstract or to the main body. We have however responded to each comment and made changes in the manuscript body (for some of the comments) to address the reviewer's observation as outlined below;

Survey target (patients are parents/guardian) not identified.
Intervention group who "used the intervention" unclear, since DAT intervention not yet described (perhaps a prior SMS-based service???). Unclear until author's summary provided some additional description of the intervention.

We thank the reviewer for this observation, we have made changes to clarify this. Our participants are children and adolescents, but since children are not able to express themselves the questions are addressed to parents/guardians. The abstract now reads as 'We conducted a survey to understand potential barriers of using DAT among CALHIV. Then, we tested a DAT intervention

among purposively selected participants. The DAT intervention included the use of the Wisepill device, receiving daily reminder SMS and receiving an adherence report on how they had taken medication over a month. Line number 40-44

Clear explicit statements regarding objectives not provided.

We would like to emphasise that the last sentence in the introduction describes the objectives. It reads as 'We aimed to assess needs, contents and acceptability of SMS based DAT intervention among CALHIV'. Line number 38-39

Uncertain if "automated adherence reports" was a key inclusion criteria or an endpoint, since objective of the study (adherence vs satisfaction vs barriers vs???) not identified.

This is the adherence report provided to participants after using the DAT for one month. The report is automatically generated by the device and can be accessed electronically anytime by the health care providers. This has been included in the sentence above describing the intervention. 'The DAT intervention included use of the Wisepill device, receiving daily reminder SMS and receiving an adherence report on how they had took medication over a month'. Line number 41-43

Timeline between identification/screening/survey/feedback adjustments not defined. Elements/targets of surveys/interviews not specified.

Thanks for this observation. Basically, we did as follows. Participant identification, screening, the survey and inclusion to the DAT study were performed on the same day. Just after the survey, selected participants were provided with the Wisepill devices and included in the DAT intervention which they used for one month. This is described in detail in the methodology under the study procedure section in the manuscript. Due to the limited number of words allowed, it is not possible to put details in the abstract. Line 174-175

Number of patients included in subsets not identified in results presented. Uncertain if subsets were selected prior to study conduct for analysis.

We have now included this. It now reads as 'We included 142 children and 142 adolescents in the survey and 20 of each used the intervention'. 47-48

"Patients were happy to use the device" not helpful since device and quantitative response not provided. Unclear how many patients used a device that was normally accessible to multiple individuals beyond the patient and/or parents that would have impacted desirability for neutral messaging. "adherence reports inspired good adherence" not helpful since device and quantitative response not provided.

Thanks for your observation. This is a mixed-methods study and these do rely on the qualitative data which do not merit quantification. However, we have reorganized/rewritten the results section in the abstract to incorporate these

comments. ‘Participants who used DAT mentioned to be happy to use the device. Ninety percent of them had good experience receiving reminders and agreed that SMS made them take medication… Provided adherence reports inspired participants to improve their adherence as told by parents and adolescents.’ Line 50-56

Unclear how focus groups data was collected (structured interviews vs themed response assessed by multiple observers vs ????).

Thanks for raising this. The FGD was conducted as a discussion between research team personnel (interviewer, facilitator and reporter) and the participants (6-8 in each FGD). The interviewer used an interview guide in an interactive process. We have added details in the section explaining Exit interviews, IDIs and FGDs. Line 205-211

Unclear how ARV regimen complexity was accounted for in the responses.

The responses on whether participants had taken the medication did not depend on the ARV regimen. It depended on how many times a day a participant took the medication. Most participants had one intake time per day while some twice a day. For every occasion, they were reminded half an hour before the intake time.

Reference to Wisepill device not helpful since no description of the technology is provided and description significantly delayed in the manuscript body.

Thank you for this comment. However, it was not clear if this is about the reference to the Wisepill device in the abstract or the manuscript body. However, the description of the Wisepill is now just after the description of the survey in the methodology section. Line 176-198

Method for adherence assessment and report format/content unclear.

We assessed adherence using three different methods, i.e. self-report, pharmacy refill counts and through DAT as indicated in appendices 1 and 2. We have added information on how adherence was calculated in Appendix 1. Appendix 5 shows an example of an adherence graph indicating the number of intakes and missed doses for one month. Line 170-171, 711-714

Method/timeline to vary messaging content not described.

Thanks for this observation. Appendices 3 and 4 details the type of content sent and how they were sent. The methodology section explains that we used one type of SMS content throughout the first week, then for the remaining 3 weeks we sent different SMS content each day. Line 188-191

Method of data distillation from interviews not provided.

We appreciate this comment, which is explained in detail under the theoretical framework and data analysis section. As described there, we conducted descriptive analysis for quantitative data and thematic framework analysis for the qualitative data. Line 213-234

Unclear at what age the children were able to provide responses in place of/in addition to the caregivers.

Thanks for raising this. We have added information to the section explaining Exit interviews, IDIs and FGDs. Line number 206-207

Author summary: unclear what functionality is provided by Wisepill and what was additional intervention by the study team?
Content and format of reminder messages unclear from present description ("how they took medication over a month" is insufficient). Schedule of message content/intensity not described (except "over time").
Intent to relate adherence feedback to adherence behavior not described in the methods, nor are the data provided to support the statement "Adherence feedbacks motivated good adherence behavior".

We are grateful to the reviewer for these very important observations. We have restructured and rewritten the author's summary according to the reviewer's comments.
We have added some information to clarify the intervention in this section. The Wisepill device created the adherence feedback report in the form of a graph which the study team discussed with the participants after they had used the device for one month.
About the schedule and content of the SMS, we changed it to describe that the SMS were sent daily, 30 minutes before the intake time and one hour post the missed dose. Since this is a summary it is not possible to give all the details of SMS contents which are well described under the methodology section.
We have re-phrased the sentence "Adherence feedback motivated good adherence behaviour" ***to 'Provided adherence reports inspired participants to improve their adherence. Line 67-77***

Sample size-unclear which of the objectives/study questions/endpoints were utilized, and no hypothesis testing described that would require such determination. Other than convenience sampling, uncertain how subgroup sizes were determined.

We thank the reviewer for this observation. The main objective of the overall study was to assess barriers to using digital tools among children and adolescents living with HIV. Hence, our sample size calculation (N=142) was based on this objective. There is no knowledge about the exact percentage experiencing such barriers. Therefore, the arbitrary percentage of 50% was used. We added more questions to the survey to investigate our objectives.

Data summary as percentages with small sample sizes (n=20) might be misleading.

We agree with the reviewer on the limitation of our small sample size. We have, however, included qualitative data to elaborate the quantitative data.

Narrative data results generally provided in tables, so can be abbreviated (and potentially combined with discussion) to reduce length.

We thank the reviewer for this observation. We have summarized the survey results in a table format. This is now presented in Table 1.

Table data should consider also providing summary statistics for the combined (children, adolescent) columns.

We thank the reviewer for this comment. However, depending on the nature of the data presented we find it hard to combine the columns for children and adolescents. Hence, we have decided to keep the separate tables.

Strengths/limitations-reconsider organization, since both are mixed within narrative instead of ordered.

Thank you. We have reorganized the strengths and limitations section. We have first described the limitations and then the strengths. Line 474-490

General Impression-this is a pilot study which explores the feasibility (but not effectiveness/efficacy) and the potential messaging content to progress to a clinical trial (intent as stated in the manuscript). I do believe the findings are an important guide to developing/optimizing existing DAT. If published, it should be significantly shortened to capture description of major design elements and thematic findings.

We thank the reviewer for this highlight. We have worked on the manuscript to make a better version.

Additional comments:
We thank the reviewer for the additional comments. We have worked on them and responded accordingly as highlighted in each point below.

Unclear if SMS messaging directed at the patient or the parent/guardian, and how that may differ among those of different age groups. Uncertain if adherence reports directed to the patient or the healthcare workers.

Thanks for raising this concern. For children aged 0-14 years, SMS were sent to their parents/guardians and adolescents without personal phones. Appendix 3 and 4 indicate the different SMS schemes for children and adolescents respectively. This has been added to the section describing the DAT, line number 180-181. Adherence reports were directed to participants (children's parents/caregivers and adolescents).

Methods (p6) Explicit statement of primary and secondary objectives would greatly facilitate understanding/evaluation of methods and statistical hypothesis testing (including sample size estimates) employed for select endpoints.

Thanks for this observation. We have added a workflow diagram (Figure 1) to explain this.

Study population-Eligibility of ART for 6mo-same or variable regimens can impact compliance. Also, children (0-14yr) responses from caregivers (not patients) while adolescents responded independent of caregiver intervention, so source of perception varied between the two groups.

Thanks for this valuable point. We have added a section about this in the discussion. Line 400-402

Survey (p7)-method of determination of adherence levels not stated.

Thanks, and we regret this omission. We have added this information in the survey section Line 170-171. Also, in appendices 1 and 2 we have added information on how the adherence levels were calculated.

Sampling for DAT: method to assure diversity in three variables (age, adherence levels and facilities) not described. (independently or in combination).

Thanks for this comment. We did a purposive selection of participants to make sure we got those with different age groups, coming from different health facilities and having low adherence levels. Each variable was assessed independently.

DAT intervention: description of the method and its functionality provided after the method of its use was provided. Method of determining adherence levels not addressed. Intervention as described includes DAT + counseling. Unclear how the behavioral model was different between children and adolescents.

Thanks for raising this. We have reorganized the DAT section. As mentioned above, the methods of determining adherence levels have been added in the survey and DAT section. Also, we included calculations under the title of Appendix 1.

The Trans theoretical model (TMM) of behavioural change was used only for the intervention (the counselling). We could not measure behavioural change since this was a pilot study to assess acceptability and the counselling section was conducted once at the end of the one month. However, we can measure behavioural change in our ongoing trial.
We modified the TMM to be applied the same to children and adolescents*. *Children who were aware of their HIV status joined their parents/caregivers during the feedback section. Line 192-198

Exit interviews-unclear how questionnaire on adherence was used adjunctively or in place of DAT-collected data. Unclear how responses quantified/characterized by interviewers.

Thanks for raising this. DAT collected data was used in calculating DAT adherence. Then the structured questionnaire used during the exit interview

was used to calculate adherence through the pharmacy refill and self-report methods. i.e. the date and number of dispensed, swallowed and missed pills. DAT adherence was calculated as (number of intakes)/ (number of days the device was in use) x 100%. Line 711-714

Tables. Include definitions for abbreviations (such as IQR).
This is included line 216.

Table 1. HIV disclosure and sharing of phone data do not account for the total population in that category
Table 2. Total participants in each category not described. Graphical feedback method described in table not described in methods. "Network" capitalized

We thank the reviewer for these correction comments. We have corrected and added the information as needed. However, the graphical feedback method is described within the last paragraph of the DAT intervention section in the methodology. Line 192-198

Affective attitude-discussion regarding "helpful tool" should be moved to discussion section. Uncertain how many participants attended boarding school (not described). Uncertain how selected quotes were chosen or whether the intent is to communicate usual or unusual observations.

We thank the reviewer for these comments. We also moved the sentence describing the intervention as a helpful tool for storage of medication to the discussion section. Line 413-416
Unfortunately, we did not record how many participants attended boarding school in the survey or the exit interview questionnaire. The findings on the perception of the intervention among this group came up during qualitative data collection through the in-depth interviews and focus group discussions. Quotes were selected to illustrate the qualitative results. They were selected depending on how well they fit in the described Theoretical Framework of Acceptability (TFA) construct.

Reviewer #2: Overall : The authors have attempted to provide deeper understanding the needs and requirements of SMS based medication adherence reminders in Children and adolescents living with HIV. Digital adherence tools and utility of text based reminders are required for viral suppression, the authors have attempted to share the learnings of what is working and not.

Major Issues:

The manuscript is very lengthy and lack cohesiveness and clarity. The quantitative and qualitative components needs to be appropriately explained and currently it needs additional clarity.

We thank the reviewer for the comments on the manuscript. We have worked and re-organized the quantitative and qualitative findings to make them clear.

1. Abstract: the methods needs to be clear and succinct. Currently, it is confusing to comprehend the methodology and steps.

We thank you the reviewer for this comment, we have re-written the methods section to make it clear. Line 40-46

2. Abstract: the results stating 93% of adolescents and 83% of children's parents were interested in receiving reminders. What is the total N or denominator 284 or 40 who were in intervention?
This refers to data from the survey in which the denominator is 142 (for each i.e. 142 children and 142 adolescents). For this variable, denominator were those had indicated to often forget to take their medication (table 1). However, we have re-placed this finding with another in the results section in the abstract. Line 47-50

3. Abstract: The percentages does not mention n and higher values can be interpreted differently. The results needs to be updated with apt numbers which adds more novelty and effectiveness for the readers.
We have worked on this section. It currently reads as 'We included 142 children and 142 adolescents in the survey and 20 of each used the intervention. Eighty five percent (121/142) of surveyed participants indicated they would like to receive reminder SMS. Most of them; 97/121(80%) of children and 94/121(78%) of adolescents would prefer to receive daily reminders. Participants who used DAT mentioned to be happy to use the device. Ninety percent of them had good experience…….'Line 47-53

4. Abstract: Language like "Adherence reports inspired good adherence" is difficult to comprehend
We have corrected this sentence to 'Provided adherence reports inspired participants to improve their adherence.' Line 53-54

5. Abstract: Conclusion statement "We will implement this in a clinical trial to assess effectiveness in improving adherence" needs to be edited and the studies take home message should be highlighted in summary line.
Thank you for this comments, we have changed the conclusion statement to 'This study showed that the proposed DAT is acceptable and provided insight of the needed SMS contents among CALHIV' Line 57-58

Methodology:
The methods is very long and confusing. The methods needs to written in a comprehendible way for authors to follow the study logic and feasibility. Need a workflow consort or a diagram explaining the processes and steps taken to facilitate user understanding.
We thank the reviewer for this constructive and guiding comments. We have summarized the methodology section and included a flow chart diagram to better explain the study steps. Line 161-162

Not sure what is the steps or operation flow in the assessment of DAT. There is a lot of surveys, and interviews but not clear why it was chosen or performed to understand the DAT effectiveness.
The survey was conducted before including participants to the DAT intervention. It was conducted to understand potential barriers of using DAT among children and adolescents living with HIV. Purposively selected participants were then included in the DAT study which they used for one month. Following that, we conducted exit interviews in-depth interviews and focused group discussions to assess acceptability of the intervention including preference of the SMS contents. This is now well explained in the methodology section where we have also included a workflow diagram. Line 161-162

The intervention sampling states participants were purposively selected, does it mean they were selected for any given reason? Then is there a selection bias?

Purposive sampling was used to select participants for the DAT intervention. We purposively selected participants to get heterogeneity in age, adherence levels and levels of health facilities. Line 177-178

Results:
The education back ground of the participants. Not sure the reason or impact of children education in DAT intervention, as the caregiver is has the access and control of the medications.

Thanks for raising this. Table 1 indicates education background for surveyed participants (N=142 for both children and adolescents) and this group have not used the DAT yet. Since we presented the educational background for adolescents, we think it is best to also indicate the same for children. Even though yes, for most of the children it is the caregivers taking the main role in disease management.

Need more clarification of self-reported adherence versus Pharmacy refill and DAT reported adherence metrics. How the score has been calculated. Seems from the qualitative IDI's the participants are reminded about the delay in taking the medication on time. Is the score updated when the participants take the medication after the second text message. Appendix 1: The <18 population has some 2 year and 4 year old where adherence is 100% across the table and for some DAT adherence shows variations. Not clear about the interpretation.

We thank the reviewer for this comment. We have added calculations of different adherence methods in the appendix 1.
The DAT adherence score is calculated as the (sum of intakes i.e. early + late + on time intakes)/ (number of days the device was in use) x 100%. If participants took their medications after the first or the second reminder, it was recorded and included in the calculation of the final DAT adherence score.
The aim of Appendix 1 and 2 is to indicate participant characteristics for those include in the DAT intervention. The difference between DAT adherence, self-report and pharmacy refills adherence could be due to that DAT adherence not being accurate due to technical or network challenges or not being use. But

also, high adherence levels by self-report and pharmacy report could be due to social desirability bias. However, it was not the objective of this paper to assess the effectiveness of DAT in estimating adherence levels.

The qualitative section of the paper has the most information about the device and text message. The manuscript should focus on this section as it provides novel information about what is working and what is not. Especially DAT interventions and feasibility shows the value of DATs to improve medication adherence. The results section needs attention to make a cohesive and well explained study findings.

We thank the reviewer for this comment. We have worked on the results section and made it more cohesive and well-explained.

Discussion: the section is very brief and requires additional comparison for the qualitative themes and quantitative findings.

We thank the reviewer for this comment. We have also worked on the discussion and added a comparison with other studies.

Overall the manuscript required a thorough revision and changes to make it brief and well articulation. The sample size of 40 and generating descriptive values of high percentages needs to be addressed.

We have worked on the manuscript to make it shorter and well-articulated. We agree with the reviewer on our small sample size and mentioned that as one of the limitations of this study. However, the descriptive data is followed by explanations from the qualitative findings.

While revising your submission, please upload your figure files to the Preflight Analysis and Conversion Engine (PACE) digital diagnostic tool, https://pacev2.apexcovantage.com/. PACE helps ensure that figures meet PLOS requirements. To use PACE, you must first register as a user. Registration is free. Then, login and navigate to the UPLOAD tab, where you will find detailed instructions on how to use the tool. If you encounter any issues or have any questions when using PACE, please email PLOS at figures@plos.org. Please note that Supporting Information files do not need this step.

We have uploaded figures 1 and 2 to the preflight analysis and PACE.

Again, we thank the editor and reviewers for their valuable comments on our manuscript. We have attached the version with track changes and a clean version of the manuscript. We hope the manuscript has significantly improved and can now be considered for publication.

Sincerely,
Iraseni Swai
